# Solitary Pubic Osteochondroma Presenting With Sexual Discomfort in a Young Adult Male: A Case Report

**DOI:** 10.7759/cureus.94314

**Published:** 2025-10-10

**Authors:** Sandeep Kumar Yadav, Sammarjanki Rymbai, Vimal Prakash, Abhay Elhence, Sudeep Khera

**Affiliations:** 1 Orthopedics, All India Institute of Medical Sciences, Jodhpur, Jodhpur, IND; 2 Pathology, All India Institute of Medical Sciences, Jodhpur, Jodhpur, IND

**Keywords:** benign tumors, bone tumour, orthopaedic oncology surgery, orthopaedics surgery, solitary osteochondroma

## Abstract

We report the case of a young adult male in his early twenties who presented with a gradually enlarging swelling in the right groin extending to the base of the penis, associated with discomfort during erection, and pain after prolonged sitting. Clinical examination revealed a firm, immobile bony swelling in the pubic region. Radiographs and MRI demonstrated a large bony lesion measuring approximately 60 × 73 × 53 mm arising from the right pubic bone and inferior pubic ramus, with continuity of the cortex and medulla and a cartilage cap measuring about 9.6 mm. The lesion was closely abutting the penile base but without surrounding soft tissue edema. Histopathological examination of a core biopsy confirmed an osteochondroma without malignant features.

The patient underwent complete excision of the mass using Ludloff’s approach, including removal of the cartilaginous cap. He resumed walking on the first postoperative day and remained symptom-free at one-year follow-up, with no evidence of recurrence. This case highlights a rare presentation of solitary pubic osteochondroma manifesting as sexual discomfort in a young adult male. It underscores the importance of clinical suspicion, detailed imaging for characterization and surgical planning, and timely excision to achieve symptom relief and prevent long-term complications.

## Introduction

Osteochondromas are the most common benign bone tumors, accounting for about 20-50% of all cases [1]. They are thought to arise from small cartilage nodules within the periosteum [1]. These tumors can be pedunculated or sessile, and the most commonly affected regions are the distal femur, proximal tibia, and proximal humerus [2]. They may occur as solitary lesions or as part of hereditary multiple exostoses (HME), which is associated with mutations in the *EXT1* and *EXT2* genes [2].

Pelvic osteochondromas represent approximately five percent of all osteochondromas, with pubic ramus involvement being particularly rare and sometimes presenting as an inguinal swelling [3]. Surgical excision is generally indicated when patients develop pressure-related symptoms, such as compressive neuropathy or obstruction of the urethra, bladder, or other vital structures [4,5]. Several reports have also described associated urogenital symptoms [6-11].

This case highlights an unusual presentation of solitary pubic osteochondroma manifesting as sexual discomfort in a young adult male. It underscores the importance of clinical suspicion, appropriate imaging for characterization, and timely en-bloc excision to relieve symptoms, prevent long-term complications, and achieve a favorable functional outcome without recurrence.

## Case presentation

A young male in his twenties presented to the outpatient department with a swelling in the right groin extending to the base of the penis. The swelling had gradually increased in size over the past two years, and the patient sought medical attention due to discomfort during erection and pain after prolonged sitting, expressing concern about his sexual health. On examination, a firm, bony-hard swelling fixed to the underlying bone and measuring approximately 8 × 10 cm was noted in the right pubic region, extending to the root of the penis. The overlying skin was free and mobile, and no other swellings were identified elsewhere on the body.

Investigations

**Figure 1:**
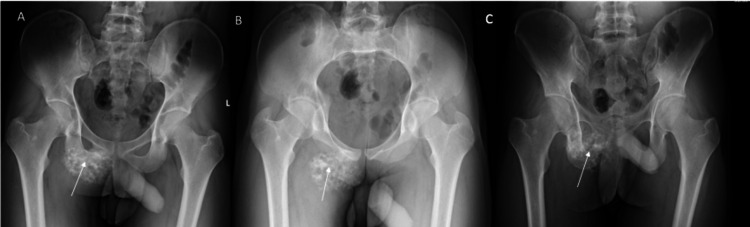
Radiographs Plain radiographs of the pelvis showing a lesion arising from the pubic bone (arrows). (A) Anteroposterior view, (B) Pelvic inlet view, (C) Pelvic outlet view.

Plain radiographs of the pelvis demonstrated a bony mass arising from the right pubic bone and inferior pubic ramus (Figure 1).

**Figure 2:**
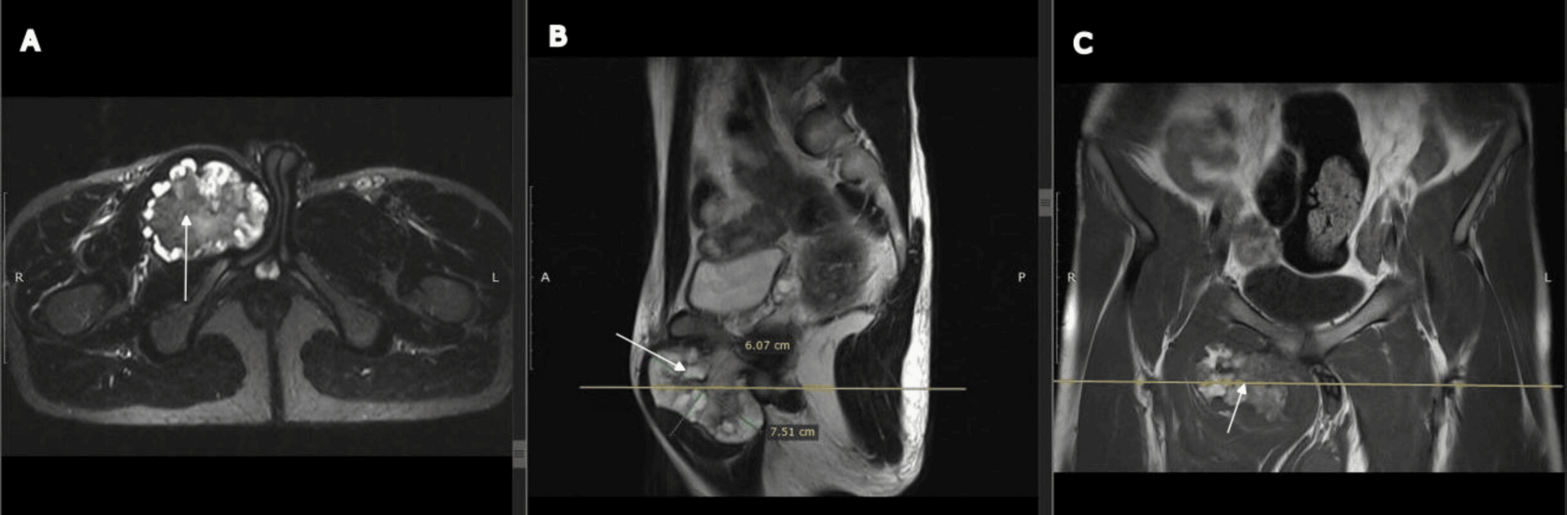
MRI images of the patient Magnetic resonance imaging showing a large bony lesion (arrows) arising from the anterior cortex of the right pubic bone and the adjacent portion of the inferior pubic ramus, with continuity of the parent medulla into the lesion. A cartilage cap measuring 9.6 mm surrounds the mass. The lesion abuts the base of the penis and extends between the right adductor muscles with preserved fat planes. (A) Axial cut, (B) Sagittal cut, (C) Coronal cut.

Magnetic resonance imaging revealed a large bony lesion measuring 60 × 73 × 53 mm in transverse, craniocaudal, and anteroposterior dimensions. The lesion originated from the anterior cortex of the right pubic bone and the adjacent portion of the inferior pubic ramus, with continuity of the parent medulla into the mass. A cartilage cap measuring 9.6 mm was noted. The lesion abutted the base of the penis and extended between the right adductor muscles with preserved fat planes. No surrounding soft tissue edema was observed. The hip joints, sacroiliac joints, and pubic symphysis were normal (Figure 2).

**Figure 3:**
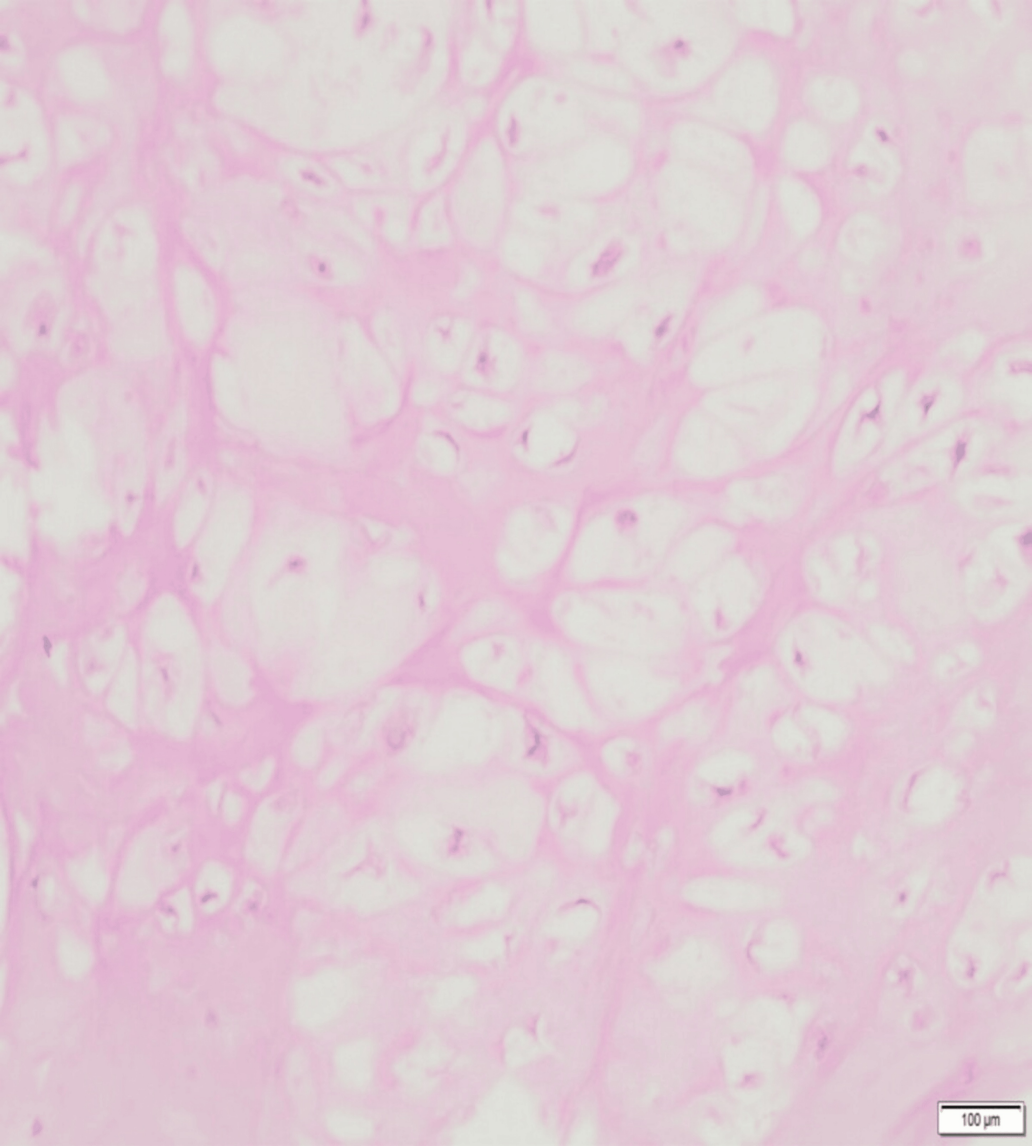
Histopathology image Histopathological section showing fragments of tumor composed of hyaline cartilage contiguous with mature bone. The tumor is moderately cellular without nuclear pleomorphism, necrosis, or increased mitotic activity, confirming the diagnosis of osteochondroma. (H&E stain, scale bar = 100 μm)

A core needle biopsy was performed under image guidance. Histopathological examination confirmed the diagnosis of osteochondroma (Figure 3).

Treatment

**Figure 4:**
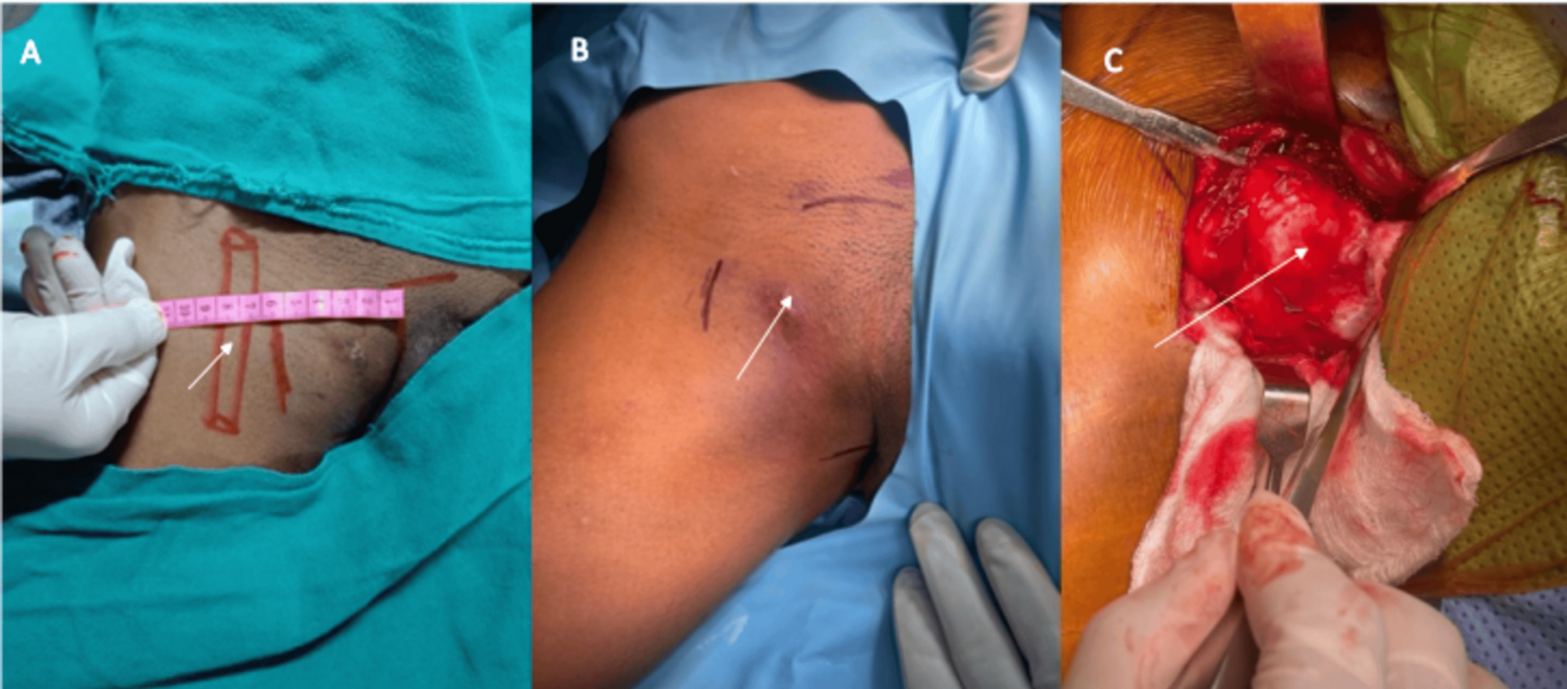
Intra-operative images Intraoperative images of the lesion (arrows). (A) Clinical photograph showing the proximity of the swelling to the femoral artery. (B) Clinical photograph highlighting the most prominent part of the tumor. (C) Intraoperative view after surgical exposure showing the cartilaginous cap of the lesion.

**Figure 5:**
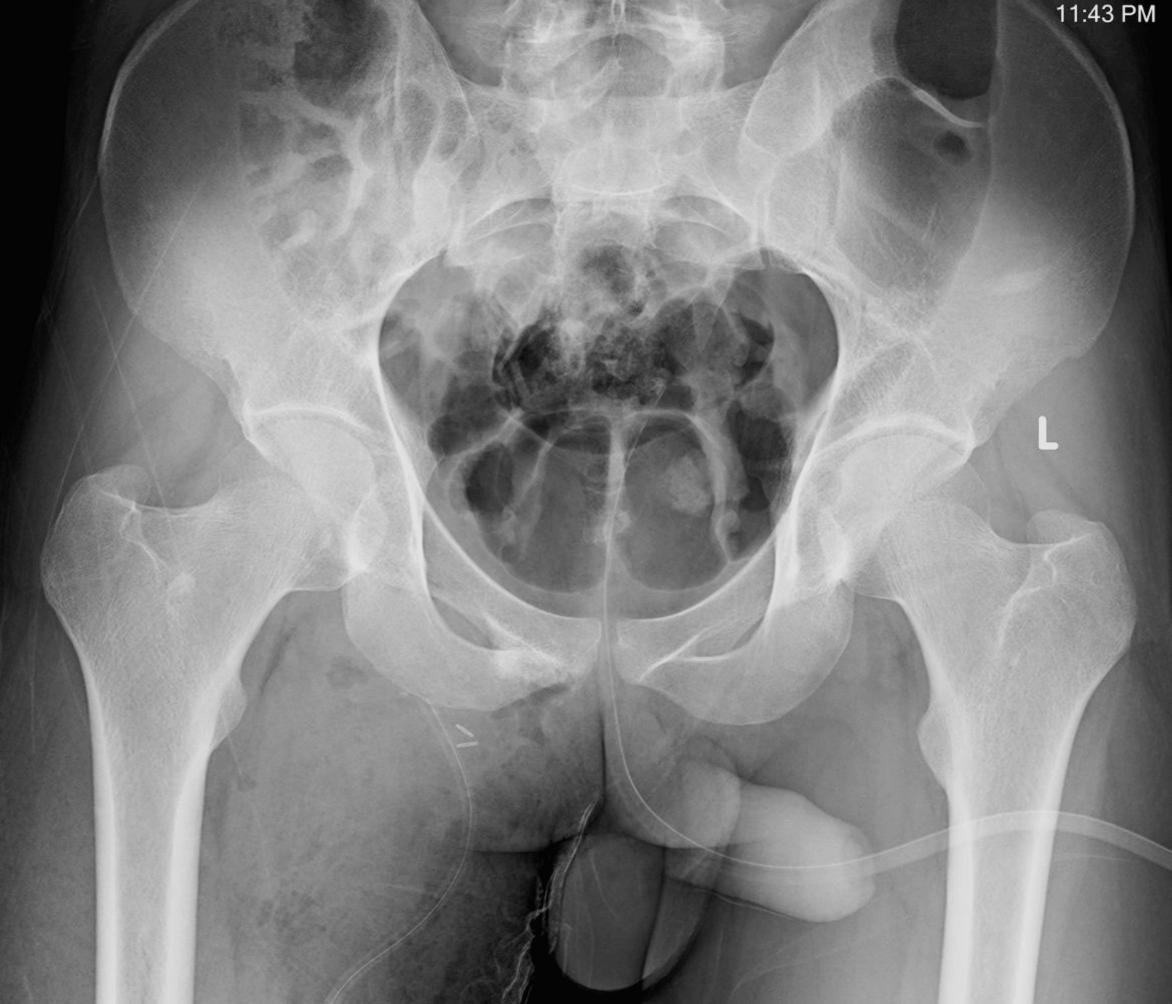
Postoperative radiograph Postoperative radiograph of the pelvis showing complete excision of the osteochondroma with restoration of normal bony contour.

Excision of the osteochondroma of the pubis was planned, and the procedure was carried out using Ludloff’s approach. The entire mass, including the cartilaginous cap, was excised en bloc (Figure 4). Patient tolerated the procedure well without intraoperative complications. The postoperative pelvic radiographs demonstrated successful excision of the osteochondroma, with restoration of the normal bony anatomy (Figure 5).

Outcome and follow-up

The patient was able to ambulate on the first postoperative day. At one-year follow-up, he had returned to his routine daily activities with normal sexual function and no evidence of recurrence.

## Discussion

Osteochondromas are the most common benign bone tumors. They are often considered developmental abnormalities of the growth plate rather than true primary bone neoplasms [3]. Commonly affected sites include the metaphysis of long bones such as the distal femur, proximal tibia, and proximal humerus, with less frequent occurrences elsewhere. Males are more commonly affected than females, with a reported ratio of 1.6-3.4:1 [4]. Osteochondromas may also be associated with multiple hereditary exostoses linked to mutations in the *EXT1* and *EXT2* genes. Trauma and inflammation have been suggested as additional contributing factors [3,4].

Most osteochondromas are incidental findings, but some cause symptoms such as cosmetic deformity or pain. Symptoms often arise during the growth phase and typically subside after skeletal maturity. Compression of adjacent neurovascular structures or malignant transformation to chondrosarcoma, though rare, are additional indications for removal [6,7]. A cartilage cap exceeding 2 cm in thickness after skeletal maturity is considered a potential indicator of malignancy [2]. Our patient, despite having swelling for years, delayed seeking care due to the social stigma of genital examination and later presented with dyspareunia.

Preoperative imaging is essential for assessing lesion extent and differentiating benign from malignant tumors [2]. In our case, X-rays and MRI were used to determine lesion size and cartilage cap thickness. A cartilage cap exceeding 2 cm post-maturity indicates possible malignancy; in our patient, the cap measured about 1 cm. Given this borderline thickness and the patient’s anxiety, a core biopsy was performed, confirming osteochondroma without malignant transformation. Although bone scans are often advised to exclude similar lesions elsewhere, we opted for a skeletal survey in this case to rule out multiple lesions.

According to the literature, pelvic osteochondromas account for approximately 5% of all osteochondromas [2,9]. Osteochondroma near the pubic symphysis is rare. Reported cases include pelvic osteochondromas causing nerve root compression or bladder outlet obstruction, necessitating removal [10,12]. Song et al. reported hematuria caused by osteochondroma of the pubic symphysis [7]. Similarly, an osteochondroma of the superior pubic ramus presenting as a cosmetic deformity has been described [3]. Hoshimoto reported sexual disturbance caused by osteochondroma of the pubic bone in an adult female [11]. Mnif et al. reported pubic exostosis causing urethral compression and sexual discomfort in a man with multiple exostoses disease [10]. In our case, the patient had discomfort during erection and was concerned about his sexual health. Surgical excision of the lesion was performed, and the patient remains symptom-free with no recurrence at one-year follow-up.

## Conclusions

Solitary osteochondroma of the pubic bone is rare and may present with atypical symptoms such as sexual discomfort or pressure on adjacent structures. Osteochondromas should be considered in the differential diagnosis of groin or genital swellings. Accurate diagnosis requires thorough clinical evaluation and appropriate imaging, including radiographs and magnetic resonance imaging, to assess lesion size, cartilage cap thickness, and relation to surrounding structures. Core needle biopsy may be necessary to exclude malignant transformation. En bloc surgical excision, as performed in this case, allows complete removal and effective symptom relief. At one-year follow-up, the patient remained asymptomatic with no evidence of recurrence. Early recognition and timely surgical management of such uncommon pelvic osteochondromas can lead to excellent functional and clinical outcomes.
